# Identification of Novel Human Monocyte Subsets and Evidence for Phenotypic Groups Defined by Interindividual Variations of Expression of Adhesion Molecules

**DOI:** 10.1038/s41598-020-61022-1

**Published:** 2020-03-10

**Authors:** F. Merah-Mourah, S. O. Cohen, D. Charron, N. Mooney, A. Haziot

**Affiliations:** 1 INSERM U976, Hôpital Saint-Louis, Paris, France; 2 Institut de Recherche Saint-Louis, Université Paris-Diderot, Paris, France

**Keywords:** Inflammation, Monocytes and macrophages

## Abstract

Monocytes contribute to immune responses as a source for subsets of dendritic cells and macrophages. Human blood monocytes are classified as classical, non-classical and intermediate cells. However, the particular functions of these subsets have been hard to define, with conflicting results and significant overlaps. One likely reason for these ambiguities is in the heterogeneity of these monocyte subsets regrouping cells with divergent functions. To better define monocyte populations, we have analysed expression of 17 markers by multicolour flow cytometry in samples obtained from 28 control donors. Data acquisition was tailored to detect populations present at low frequencies. Our results reveal the existence of novel monocyte subsets detected as larger CD14^+^ cells that were CD16^+^ or CD16^neg^. These large monocytes differed from regular, smaller monocytes with respect to expression of various cell surface molecules, such as FcR, chemokine receptors, and adhesion molecules. Unsupervised multidimensional analysis confirmed the existence of large monocytes and revealed interindividual variations that were grouped according to unique patterns of expression of adhesion molecules CD62L, CD49d, and CD43. Distinct inflammatory responses to TLR agonists were found in small and large monocytes. Overall, refining the definition of monocyte subsets should lead to the identification of populations with specific functions.

## Introduction

Monocytes, which are mostly precursors of some macrophage and dendritic cell populations, have been hard to divide into populations with clear-cut inflammatory and immune functions. This may be due in part to the high number and complexity of phenotypes present in monocyte populations.

A classification of human blood monocyte subsets based on the expression of CD14 and CD16 cell surface receptors was proposed^1^ and refined over the years^2,3^. It consisted initially of two populations described as “classical” monocytes, which express CD14 but no CD16, and “nonclassical” monocytes with low CD14 and strong CD16 expression. Further analysis identified CD14^+^CD16^+^ monocytes with expression of CCR5 and intermediate expression of receptors divergently expressed in the two other subsets (e.g. CCR2 and CX3CR1^4^. This additional subset was identified as “intermediate”^5^. Evidence for this third subset was confirmed in transcriptome analysis^6–10^.

Despite progress in phenotypic analysis, immune functions associated with monocyte subpopulations in the steady state remained ill defined. Marked functional redundancies between the sub-populations were found, and contradicting results in the literature added to the puzzling difficulties in assigning functions to specific populations^11–13^. Thus, intermediate monocytes were described as the major source of pro-inflammatory cytokines upon stimulation^14,15^. In contrast, non-classical monocytes were also described as the most inflammatory monocytes^3,13,16^. Similarly, anti-inflammatory cytokine secretion was alternatively found high in intermediate^13^ or in classical monocytes^6,11,17^.

Therefore, production of pro-inflammatory and anti-inflammatory cytokines upon activation, a hallmark property of monocytes, remains hard to ascribe unambiguously to given subsets.

In this study, we stringently assessed the phenotypic heterogeneity of human blood monocytes by multicolour cell surface labelling, flow cytometry analysis, and unsupervised detection of clusters and analysis of their phenotypes. To look for conserved phenotypes, the analysis was extended to 28 healthy Caucasian donors. Results identified novel populations of monocytes with unique morphologic and phenotypic characteristics, and with distinct inflammatory responses to TLR agonists. Although monocyte populations had heterogeneous phenotypes among healthy donors they could nonetheless be resolved into phenotypic groups based on interindividual variations of expression.

## Results

### Identification of populations of monocytes in human healthy donors

**Figure 1:**
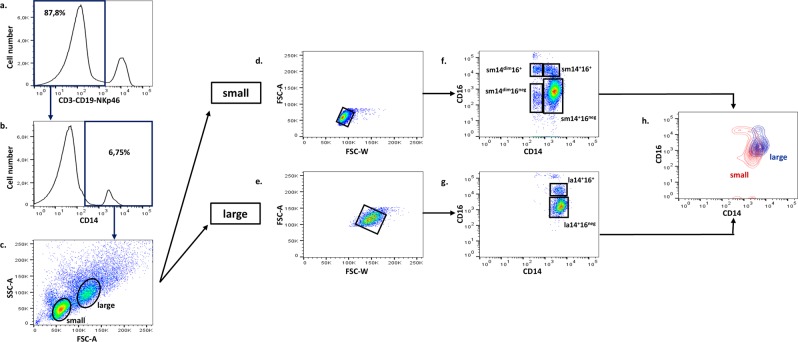
Gating strategy and identification of monocyte subpopulations. PBMC were separated and stained with antibodies directed at various cell surface molecules (Supplementary Table S2). Lineage markers were used to exclude lymphocyte subsets (**a**) and CD14+ cells were selected (**b**). Forward and side scatter identified small and large clusters of monocytes (**c**). After exclusion of doublets from each cluster^49^ (**d**,**e**), the expressions of CD14 and CD16 were analysed in gated cells (**f**,**g**). The higher expression of CD14 in large monocytes is shown in panel h where the profiles of CD14 and CD16 expressions in large (blue contours) and small monocytes (red contours) were overlaid. A substantial part of la14^+^16^neg^ monocytes had a higher CD14 expression than sm14^+^16^neg^ cells, and almost all la14^+^16^+^ monocytes expressed more CD14 than sm14^+^16^+^ cells. Data presented were obtained from one representative donor.

In order to further define human blood monocyte populations, we have analysed labelled PBMC from 28 healthy individuals by flow cytometry (Supplementary Table S1). Settings, which were chosen to achieve a comprehensive study of monocyte populations, included an optimized procedure to reduce non-specific labelling, the acquisition of 1 × 10^6^ events to analyse rare populations, and the exclusion of non-myeloid cell lineages (CD3^+^, CD19^+^, and NKp46^+^ (CD335) cells) as initial step (Fig. 1a). CD14 expression was considered a necessary condition for inclusion in monocyte populations (Fig. 1b). In PBMC analysed in these conditions, CD14^+^ cells formed two main clusters according to their size and granularity, as shown in Fig. 1c. In addition to a main cluster representing 78.4 ± 14.0% (mean ± SD, n = 28) of CD14^+^ cells and appearing to correspond to the commonly defined monocyte population, a distinct cell population was identified. This clearly visible set of larger cells was present in all donors and constituted 8.8 ± 6.0% of CD14^+^ cells. Cells in this cluster were named large monocytes (la) in contrast to the denser set of smaller CD14^+^ cells, thus named small monocytes (sm). After exclusion of doublets in each subset (Fig. 1d,e), the expressions of CD14 and CD16 were analysed in these two populations. To show that large monocytes did not result from density gradient separation with Ficoll, monocytes were analysed in whole blood. After exclusion of lymphocytes and granulocytes, small and large monocyte populations were clearly identified in CD14^+^ cells (Supplementary Fig. S1).

In CD14/CD16 plots, small monocytes were divided into four populations (Fig. 1f). A major CD14^+^/CD16^neg^ population consisted of 77.2 ± 13.0% of this cell cluster and were named sm14^+^16^neg^. Populations of CD14^+^/CD16^+^ cells (4.4 ± 2.3%) named sm14^+^16^+^, and of CD14^dim^/CD16^+^ cells (3.2 ± 2.6%) named sm14^dim^16^+^ were also identified in all donors. A fourth population of CD14^dim^/CD16^neg^ cells named sm14^dim^16^neg^ was identified in 16 out of 28 donors where it represented 2.4 ± 1.5% of small monocytes.

Large monocytes (Fig. 1g) were divided in two populations, a CD14^+^/CD16^neg^ population representing 79.2 ± 10.0% of large monocytes and named la14^+^16^neg^, and a CD14^+^/CD16^+^ population (10.0 ± 5.2%) named la14^+^16^+^. Thus, this newly described set of large monocytes differed from small monocytes with respect to FSC scatter and levels of CD14 expression.

**Figure 2:**
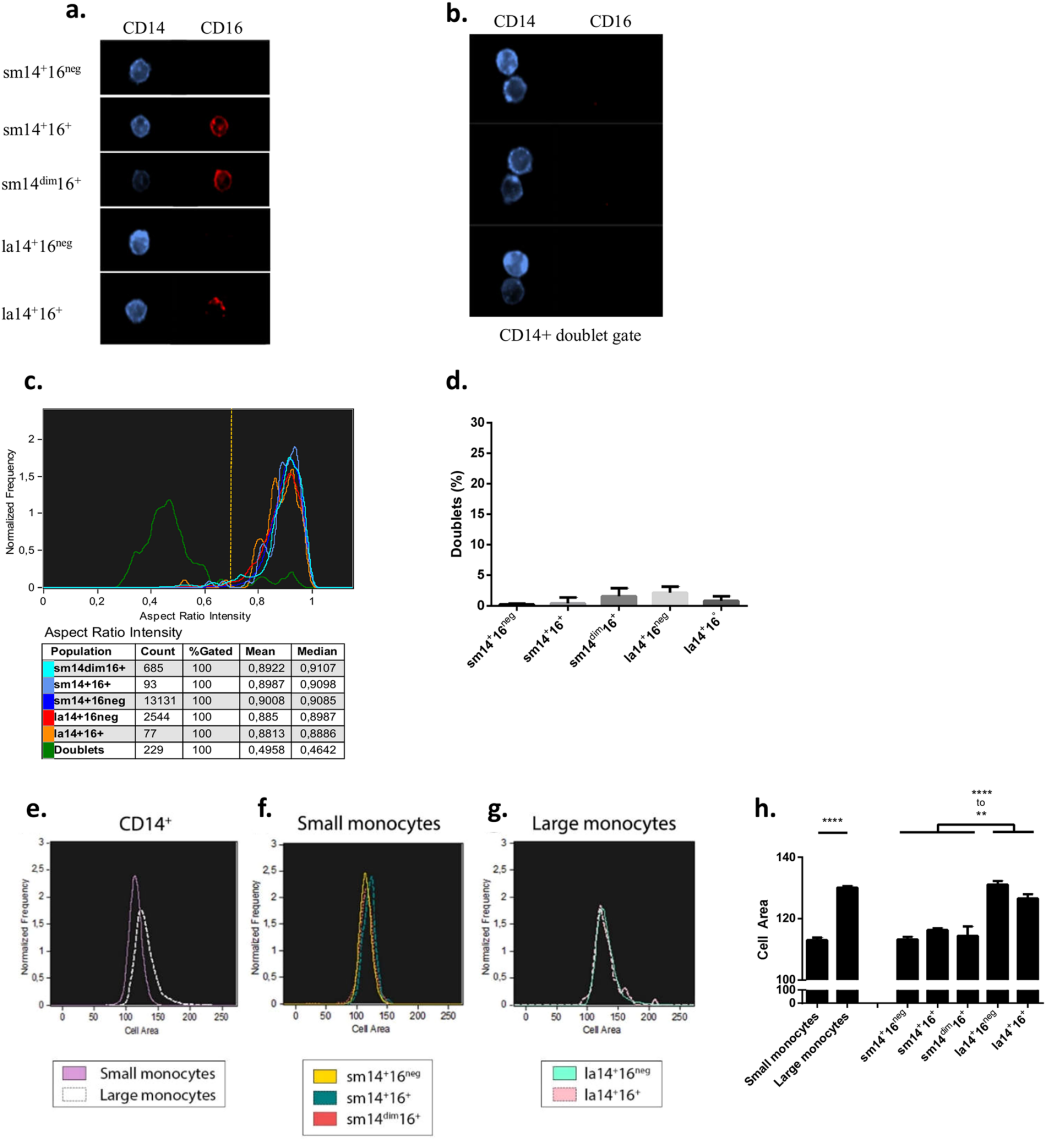
Imaging flow cytometry analysis of monocyte subpopulations. PBMC (n = 5) were stained and analysed by imaging flow cytometry (ImageStream, Amnis). After exclusion of lymphocytes, selection of CD14-positive cells, and doublet exclusion, small and large monocytes were visualized. (**a**) Representative images of small and large monocytes. (**b**) Representative images of cells in the CD14^+^ doublet gate. (**c**) Shape analysis of all events in small and large monocyte gates and in doublet gate using the aspect ratio feature (IDEAS software) in a representative donor. The ratio between the minor and major axis of each event in monocyte gates was calculated and compared to the corresponding ratio of cells in the doublet gate. A vertical bar drawn at the nadir between singlet and doublet curves (Aspect Ratio Intensity around 0.7) served as threshold to quantify singlets and doublets in each population. (**d**) Quantification of doublets present in gates used to define small and large monocyte subpopulations, calculated as events located left of the threshold bar drawn in panel c (mean ± s.d.) and expressed as percent of cells in the gate; doublets represented less than 5% of the cells in large monocyte gates, a percentage similar to that of small monocyte gates. (**e**) Cell size was determined using Area Feature (IDEAS software) with a mask delimited by CD14 expression in small and large monocytes, (**f**) in small monocyte subpopulations, and (**g**) in large monocytes subpopulations from a representative donor. (**h**) quantification of cell sizes for each subpopulation in 5 donors; sm14^+^16^neg^, sm14^+^16^+^, and sm14^dim^16^+^ monocytes had a similar size distribution, and large monocyte subpopulations la14^+^16^neg^ and la14^+^16^+^ had also very similar sizes (mean ± SEM, **p ≤ 0.01; ****p ≤ 0.0001, one way ANOVA).

To dismiss the possibility that large monocytes were doublets, PBMC were analysed by imaging flow cytometry. Large monocytes appeared as single cells (Fig. 2a) and shape analysis of cells in large monocyte gates showed that over 95% of the cells were spheroids in contrast to oblong objects present in doublet gate (Fig. 2b,c). Cell size was also analysed in imaging flow cytometry using a mask delimited by CD14 expression. Results showed that large monocytes had a significantly larger size than small monocytes (Fig. 2e,h). Cell diameters were inferred from cell size and were determined to be 12.84 microns for large monocytes and significantly larger than small monocytes that had a diameter of 11.98 microns (Supplementary Fig. S2).

Therefore, a total of six populations of monocytes were distinguished according to our gating strategy based on sequential use of lineage selection, SSC and FSC cluster analysis, doublet exclusion in gated monocyte populations, and levels of CD14 and CD16 expression.

### Phenotypic characterization of each newly defined populations of monocytes

**Figure 3:**
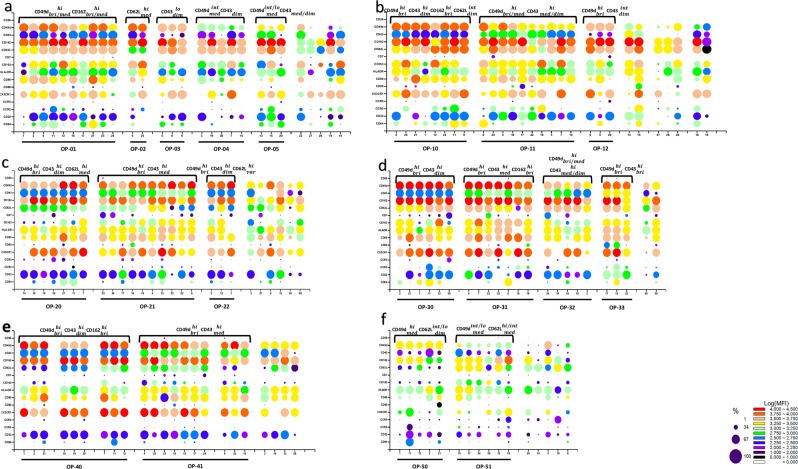
Phenotypes of monocyte subpopulations as analysed with our typing platform. PBMC from 28 donors were separated and stained with antibodies directed at various cell surface receptors such as Ig FcR (CD64 and CD32 in addition to CD16), chemokine receptors (CCR2, CCR5, and CX3CR1), antigen presentation and co-stimulatory molecules (HLA-DR, CD86, and CD80), adhesion molecules (CD62L, CD162, CD43, CD49d, and CD56). The expressions of scavenger receptor CD163^19,20^ and immunoglobulin superfamily molecule CD7^21^ were also determined (y-axis and Supplementary Table S2). Cells were analysed as described in Fig. 1. Fluorochrome-matched isotype controls were used to determine specific MFI and percentage of positive cells. Expression levels are presented as dots of colour and size reflecting MFI and percentage of positive cells, respectively, according to colour and size scales shown in legend. Monocytes subsets: (**a**) sm14^+^16^neg^, (**b**) la14^+^16^neg^, (**c**) sm14^+^16^+^, (**d**) la14^+^16^+^, (**e**) sm14^dim^16^+^ and (**f**) sm14^dim^16^neg^. Variations in the number of donors analysed in each monocyte population were due to the inability to assess the expression of markers when cell numbers were too low. For each sub-population, donors were grouped according to similar expression of markers as noted at the top of the panels and recapitulated with the OP nomenclature at the bottom of the panels.

To further characterize the identified monocyte populations, the expression of 15 cell surface receptors associated with important functions of monocytes was analysed in cells obtained from our panel of 28 donors. Thus, in addition to exclusion markers for lymphocyte populations (CD3, CD19, NKp46), monocyte population markers CD14, a LPS co-receptor^18^, and CD16, the Fc receptor FcRIII^1^, other markers analysed included chemokine receptors, CCR2, CX3CR1, and CCR5, that have been instrumental in distinguishing classical, non-classical and intermediate monocytes^16^, Fc receptors FcRI/CD64 and FcRII/CD32, antigen presentation molecule HLA-DR and co-stimulation molecules CD80 and CD86, as well as adhesion molecules CD62L/L-selectin, CD162/P-selectin ligand, CD43/leukosialin, CD49d/VLA-4, and CD56/N-CAM. The expressions of the scavenger receptor CD163^19,20^ and the immunoglobulin superfamily CD7 molecule^21^ were also determined. Marker expression for each of the six monocyte populations is presented in individual panels (Fig. 3a–f). The results confirm the widely shown preferential expression of CCR2 and CX3CR1 in CD16^neg^ (Fig. 3a,b,f) and CD16^+^ (Fig. 3c,d,e) monocytes, respectively^22^. Differences of expression between monocyte populations were identified, with higher expression of antigen presentation molecules HLA-DR (MFI) and CD86 (percent of positives) in la14^+^16^neg^ compared to sm14^+^16^neg^ (Fig. 3a,b), higher intensity of expression of adhesion molecules CD49d, CD162, and CD62L in la14^+^16^+^ compared to sm14^+^16^+^ (Fig. 3d,e). A globally lower expression of most markers characterized sm14^dim^16^neg^ monocytes (Fig. 3f). Individual variation between donors was however one of the salient feature of the analysis of expression for many markers. Remarkably, similar variations were identified in donors, which were unrelated, allowing the identification of groups among our panel of Caucasian donors. In sm14^+^16^neg^ monocytes (Fig. 3a), 15 out of the 28 donors had a high percentage of monocytes positive for CD49d and CD162 (CD49d^hi^ and CD162^hi^) with a MFI in the bright to medium range (${\rm{CD}}49{{\rm{d}}}_{bri/med}^{hi},\,{\rm{CD}}{162}_{bri/med}^{hi}$). Among this group, two donors were set apart based on ${\rm{CD}}62{{\rm{L}}}_{med}^{hi}$ expression defining profile OP-02, and three others were distinguished according to a ${\rm{CD}}{43}_{dim}^{lo}$ expression (OP-03). The remainder was named OP-01. Similarly, a second group of five donors was individualized based on a ${\rm{CD}}49{{\rm{d}}}_{med}^{int}\,{\rm{and}}\,{\rm{CD}}{43}_{dim}$ phenotype, a profile named OP-04. A final group of three donors (OP-05) was identified with the common phenotype ${\rm{CD}}49{{\rm{d}}}_{med}^{int/lo}\,{\rm{and}}\,{\rm{CD}}{43}_{med/dim}$. In these two last groups, a ${\rm{CD}}{162}_{bri}^{hi}\,{\rm{and}}\,{\rm{CD}}62{{\rm{L}}}_{bri}^{hi}$ expression was found. Five donors from this panel could not be categorized along a unique phenotype.

Similarly, the other monocyte populations could also be split according to the patterns of expression of adhesion molecules. Among large monocytes, la14^+^16^neg^ monocytes (n = 28) (Fig. 3b) were distinct from their CD16^neg^ counterpart sm14^+^16^neg^ monocytes (Fig. 3a) in their overall stronger expression of several cell surface molecules (CD163, HLA-DR, CD86, CX3CR1, and CCR2). In contrast, adhesion molecules CD162 and CD62L were in general similarly expressed in la14^+^16^neg^ and sm14^+^16^neg^. Several groups of donors were defined in la14^+^16^neg^ monocytes according to the expression of adhesion molecules CD49d and CD43 with a ${\rm{CD}}49{{\rm{d}}}_{bri}^{hi},\,{\rm{CD}}{43}_{dim}^{hi}$ phenotype (n = 8), a ${\rm{CD}}49{{\rm{d}}}_{bri/med}^{hi},\,{\rm{CD}}{43}_{med/dim}^{hi}$ phenotype (n = 10), and a ${\rm{CD}}49{{\rm{d}}}_{bri}^{hi},\,{\rm{CD}}{43}_{dim}^{int}$ phenotype (n = 3), and were named OP-10, OP-11, and OP-12, respectively. In 7 donors, atypical profiles with ${\rm{CD}}{43}_{bri}^{hi}$ (n = 2), CD43^*neg*^ (n = 3), and ${\rm{CD}}49{{\rm{d}}}_{med/dim}^{int/hi},\,{\rm{CD}}{43}_{dim}^{int/hi}$ (n = 2) were found.

In sm14^+^16^+^ monocytes (Fig. 3c), the expression of CD49d and CD162 was relatively conserved among most of the donors with a large majority of donors (20 out of 26) phenotyped as ${\rm{CD}}49{{\rm{d}}}_{bri/med}^{hi}\,and\,{\rm{CD}}{162}_{bri/med}^{hi}$. Among these, three profiles could be identified according to CD43 and CD62L expression with the following phenotypes ${\rm{CD}}{43}_{dim}^{hi}\,{\rm{and}}\,{\rm{CD}}62{{\rm{L}}}_{med}^{hi}$ (n = 7) with a low level of CCR5 expression in some donors, ${\rm{CD}}{43}_{med}^{hi}$ (n = 10), and ${\rm{CD}}{43}_{dim}^{hi},\,{\rm{and}}\,{\rm{CD}}62{{\rm{L}}}_{variable}^{hi}$ (n = 3). These profiles were named OP-20, OP-21, and OP-22. Six donors had distinct patterns of expressions of these markers and were not grouped.

la14^+^16^+^ monocytes (n = 23) (Fig. 3d) differed from sm14^+^16^+^ monocytes (Fig. 3c) with a stronger expression of adhesion molecules CD49d, CD162, and CD62L, and of CD163, and a brighter expression of CCR2 when expressed. Donors were grouped based on expression of adhesion molecules CD49d and CD43 with a ${\rm{CD}}49{{\rm{d}}}_{bri}^{hi},\,{\rm{CD}}{43}_{dim}^{hi}$ group (n = 6), a ${\rm{CD}}49{{\rm{d}}}_{bri}^{hi}\,,\,{\rm{CD}}{43}_{med}^{hi}$ group (n = 7), a ${\rm{CD}}49{{\rm{d}}}_{bri/med}^{hi},\,{\rm{CD}}{43}_{med/dim}^{hi}$ group (n = 5), and a ${\rm{CD}}49{{\rm{d}}}_{bri}^{hi},\,{\rm{CD}}{43}_{bri}^{hi}$ group (n = 3). These groups were termed OP-30 to OP-33. Two donors with atypical combinations of expression of adhesion molecules were left ungrouped (numbers 26 and 28).

Less individual variability with respect to adhesion molecules was detected in sm14^dim^16^+^ monocytes (n = 23) (Fig. 3e). A fairly consistent expression of CD49d, CD162, as well as HLA-DR, and CX3CR1 was found in these cells with the exception of four donors (on the right side of the figure) with lower expression of CD49d and/or CX3CR1. Two main patterns named OP-40 and OP-41 were identified with a ${\rm{CD}}49{{\rm{d}}}_{bri}^{hi},\,{\rm{CD}}{43}_{dim}^{hi},\,{\rm{and}}\,{\rm{CD}}{162}_{bri}^{hi}$ phenotype (n = 9) in which varying degrees of CD62L expression were distinguished, and a ${\rm{CD}}49{{\rm{d}}}_{bri}^{hi},\,{\rm{CD}}{43}_{med}^{hi}$ phenotype (n = 10) including 7 donors characterized as ${\rm{CD}}{162}_{bri}^{hi}\,{\rm{and}}\,{\rm{CD}}62{{\rm{L}}}_{med}^{hi}$. Four donors had a ${\rm{CD}}49{{\rm{d}}}_{int}^{hi},\,{\rm{CD}}{162}_{bri/int}^{hi}$ phenotype together with lower expression of CX3CR1.

The sm14^dim^16^neg^ monocyte population (Fig. 3f) could be analysed in 16 donors only, due to its varying presence among monocyte populations. Profiles of expression in this population were strikingly different from its CD16^+^ counterpart, sm14^dim^16^+^ monocytes (Fig. 3e) with low expression of adhesion molecules CD49d, CD162, CD62L and of chemokine receptor CX3CR1. Remarkably, HLA-DR was similarly expressed but this expression was coupled with scant CD86 expression in most donors. Groups were defined along expression of CD49d and CD62L with 5 donors characterized as ${\rm{CD}}49{{\rm{d}}}_{med}^{hi},\,{\rm{CD}}62{{\rm{L}}}_{dim}^{int/lo}$, and six donors as ${\rm{CD}}49{{\rm{d}}}_{med}^{int/lo},\,{\rm{CD}}62{{\rm{L}}}_{med}^{hi/int}$ named OP-50 and OP-51, respectively. Five donors remained without classification.

From these results, it appears that variations in the expression of selected markers (CD49d, CD43, CD162, and CD62L) did not occur randomly in the monocyte subpopulations and may correspond to a concerted profile of gene expression.

**Figure 4:**
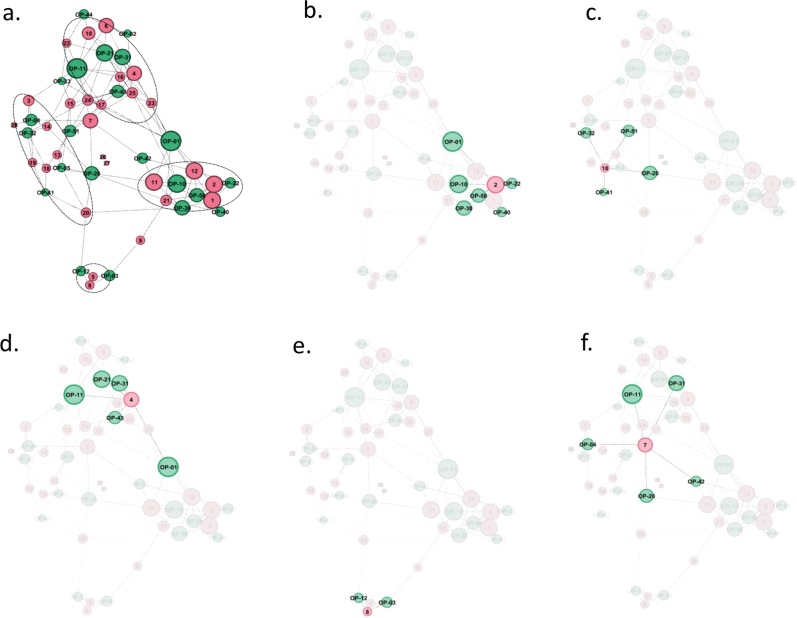
Connectivity map between donors and defined phenotypic profiles. To determine which phenotypic profiles identified in subpopulations of monocytes were more likely to be associated in a given donor, a network analysis was performed using Gephi^23^. Green dots represent phenotypic profiles (OP, Fig. 3) and pink dots represent donors. The size of the dots is proportional to the number of links. (**a**) Complete network with ovals around clustered donors as listed in Table 1; (**b**–**e**) one donor representative of each cluster with links to corresponding phenotypic profiles; (**f**) one non-clustered donor. Thus, monocyte phenotype I was composed of profiles OP-01, -10, -22 or -20, -30, -40 or -42, and -50 and was present in 5 donors (see Table 1). In monocyte phenotype II, no profile was strongly associated with la14^+^16^neg^ monocytes, with donors 3 and 14 having profile OP-11, donor 20 having profile OP-12, and donors 13, 18, and 19 having no characteristic la14^+^16^neg^ profile. However, in other monocyte sub-populations, these donors shared profiles OP-04 or -05, -20, -32, -41, and -51. Monocyte phenotype III included profiles OP-01 or -02, -11, -21, -31, and -43 or -44 with however some divergence in donors 22 (with OP-33), 23 and 25 (with OP-10). Donors 17 and 25 had profile OP-51 in sub-population sm14^dim^16^neg^ whereas no other donor had distinctive profiles in this sub-population. Monocyte phenotype IV was much less defined and included profiles OP-03 and -12 found in only two donors (5 and 8). Conspicuous in these two donors was the paucity of CD16 positive monocytes, a feature also found in donor 9. Monocyte phenotypes II and III were also linked by donors 7, 15, and 24 that shared parts of their profiles.

In an attempt to further define monocyte phenotypes across the sub-populations, we set out to determine which profiles detected in small and large monocytes were more likely to be associated in healthy donors. We used the network analysis tool Gephi^23,24^ to map the connectivity between profiles OP-01 to OP-51 based on the 28 donors analysed. As shown in Fig. 4, OP profiles formed clusters based on relations to profiles found in each donor. Four clusters were identified. They consisted of profiles from each monocyte sub-populations that are more likely to be associated in a whole monocyte population (Table 1). Overall, identification of these monocyte phenotypes in donors further reinforces the notion that monocyte expression of adhesion molecules best reflects the phenotypes of these cells in subsets within subpopulations.

**Table 1:** Clusters of donors defined according to phenotypic profiles identified in monocyte subpopulations.

Donors	OP profiles	Global monocyte phenotypes
1–2–11-12-21	01 10 20/22 30 40/42 50	I
3–13–14–18–19–20	04/05 11/12 20 32 41 51	II
4–6–10–16–17–22–23–25	01/02 10/11 21 31/33 43/44 51	III
5–8	03 12	IV
7–9–15–24–26–27–28		Non clustered

We then looked for associations between Gephi-defined clusters and donor characteristics such as age and sex. Interestingly, as shown in Supplementary Fig. S3, although the numbers were relatively small in each cluster, subjects in cluster III were older than subjects in cluster I (mean ± sd 51.6 ± 13.4 vs. 34.4 ± 9.5 years). The difference was statistically significant (p = 0.045, Mann-Whitney U test). No other age difference between clusters reached statistical significance. Sex ratios in most clusters were unremarkable (Supplementary Fig. S4). However, in cluster I females were underrepresented and males were overrepresented compared to the parent population 98% confidence interval. These results suggest that age and sex may be contributing factors in the variations of monocyte phenotypes.

### Identification of clusters of defined phenotypes within monocyte populations

Having established the overall expression of selected markers in newly defined monocyte populations, we sought to analyse the combined expression of the markers at a single cell level and to identify cell clusters with similar profiles of expression in an unsupervised manner. We used SPADE, a hierarchical analysis generating branched tree structure of related cells, followed by analysis with viSNE, which allows visualization of high-dimensional single-cell data.

#### SPADE analysis

We used SPADE^25^ for 20 markers at high resolution and specificity to generate a hierarchy of cell clusters, represented as a tree, for each donor. Analysis of side and forward scatter properties of each branch of the tree together with CD14 expression allowed us to identify branches corresponding to small and large monocytes (Supplementary Fig. S5).

This unsupervised analysis validated the individualization of small and large monocytes as subpopulations.

#### viSNE analysis

**Figure 5:**
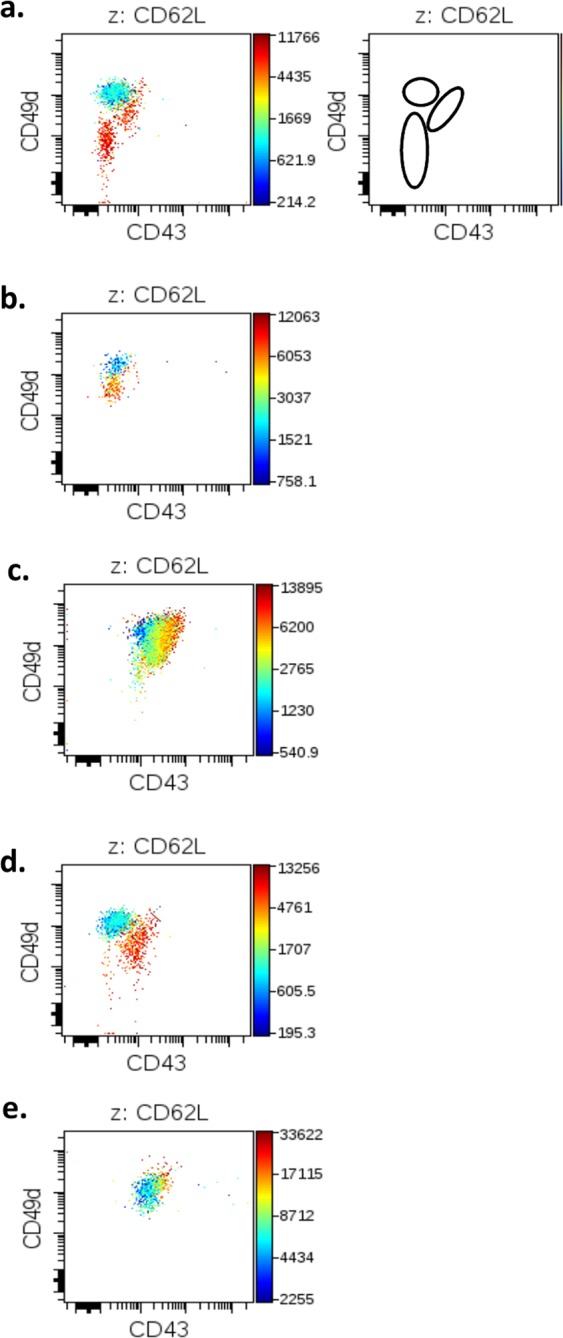
viSNE profile of sm14^+^16^+^ monocytes. Small and large monocyte populations identified with SPADE (Supplementary Fig. S5) were analysed with viSNE and specific expression profiles of adhesion molecules were identified in donors. The expression of CD43, CD49d and CD62L (x, y, z) in sm14^+^16^+^ monocytes was represented here in dot plot profiles, with CD62L expression shown according to a colour scale (right hand side axis). Profiles shown in  (**a–e**) correspond to monocyte sub-populations  a, b, c, d, e identified in Table 2. In panel **a**, a schematic representation of monocyte populations identified in profile a and that are found in various combinations in profiles  b, c, d, e is shown. Plots shown are from representative donors expressing a to e profiles.

Small and large monocyte populations identified by SPADE were subjected to analysis with the viSNE algorithm. viSNE creates 2-D plots where the expression of each marker is taken into account to determine the position of each cell^26^. Analysis of these profiles showed a large extent of variations between donors, with multiple clusters of monocytes detected. Strikingly, among these interindividual variations, common patterns were distinguishable in groups of unrelated donors. These shared features were particularly visible when data sets were visualized with selected markers, CD49d, CD43 (used in x and y axis) and CD62L (selected for MFI color-coded representation) (Fig. 5). In sm14^+^16^+^ monocytes we identified 3 subpopulations that were present in 9 donors. These three subpopulations were formed of CD43^+^CD49d^+^CD62L^hi^ cells, CD43^dim/neg^CD49d^hi^CD62L^dim^ cells, and CD43^neg^CD49d^dim^CD62L^hi^ cells (Fig. 5a) and their concomitant presence in one donor was defined as profile a. In a second set of donors (n = 5), only populations CD43^dim/neg^CD49d^hi^CD62L^dim^ cells, and CD43^neg^CD49d^dim^CD62L^hi^ were identified defining profile b (Fig. 5b). A third profile (named c, n = 4) consisted of CD43^+^CD49d^+^CD62L^hi^ cells and CD43^dim/neg^CD49d^hi^CD62L^dim^ cells together with an intermediate population in between these two and of variable CD43 expression (Fig. 5c). A d profile was found in four donors and consisted of CD43^+^CD49d^+^CD62L^hi^ cells and CD43^dim/neg^CD49d^hi^CD62L^dim^ cells as in profile c but without the intermediate cells (Fig. 5d). Finally, only one donor (donor 10) had a unique profile (named e) with a CD43^+^CD49d^hi^CD62L^dim^ major cell population and a CD43^hi^CD49d^hi^CD62L^hi^ minor cell population (Fig. 5e). Interestingly, donors regrouped according to these patterns of expression in sm14^+^16^+^ monocytes turned out to share similarities of expression for several markers in all other monocyte sub-populations. Therefore, classification of donors into profiles a to d initially defined in sm14^+^16^+^ monocytes revealed clusters of donors with shared patterns of expression across all monocytes populations (Table 2).

**Table 2:** Identification of four monocyte phenotypic profiles by viSNE analysis.

viSNE Profile	Donors	Phenotype in sm14^+^16^+^
a	2–4–8–14–15–16–19–21–25	CD43^+^CD49d^+^CD62L^hi^ CD43^dim/neg^CD49d^hi^CD62L^dim^ CD43^neg^CD49d^dim^CD62L^hi^
b	1–3–6–12–13	CD43^dim/neg^CD49d^hi^CD62L^dim^ CD43^neg^CD49d^dim^CD62L^hi^
c	7–9–22–23	CD43^+^CD49d^+^CD62L^hi^ in-between population CD43^dim/neg^CD49d^hi^CD62L^dim^
d	17–18–20–24	CD43^+^CD49d^+^CD62L^hi^ CD43^dim/neg^CD49d^hi^CD62L^dim^

From these results, it appeared that numerous populations of monocytes could be distinguished and some were shared between donors. Most importantly, many monocyte populations were present in a fraction of the donors, defining complex intertwined phenotypic groups (a, b, c, d, and e) in our panel of 28 Caucasian healthy donors. The existence of such phenotypic groups among a population of healthy donors may further refine the definition of monocytes subsets. Overlooking these variabilities might have been an impediment in the definition of functional populations among global monocyte populations or even among the three monocyte subsets so far described in the literature.

We checked whether donors’ age and sex were associated with the identified clusters. As shown in Supplementary Fig. S6, age appeared randomly distributed among the clusters and no statistically significant difference was found. In contrast, significantly different sex ratios in clusters a and b were found compared to the parent population 98% confidence interval (Supplementary Fig. S7), suggesting that profile a and b may be more frequent in females and males, respectively.

### Divergences between groups defined according to mean expression in a population versus single cell expression of multiple markers

Results presented in Figs. 3 and 5 clearly indicated that similar patterns of inter-individual variations of expression of markers, mostly adhesion molecules, were recognizable in our panel of 28 non-related donors. However, comparison of Tables 1 and 2 shows that groups of donors constituted from composite profiles of mean expression of adhesion molecules in monocyte subpopulations (Fig. 3) and combined expression of multiple markers at the single cell level (Fig. 5) do not coincide. This suggests that composite phenotypes in donors can be achieved by different combinations of single cell profiles of expression and points to the existence of intricate patterns of regulation of gene expression in this cell type. It also raises the question of which biological significance may be attached to each classification. Identification of immune cell populations with defined phenotypes has been highly instrumental to advancing the understanding of immune functions and their mechanisms. Consequently, groups based on complex phenotypes determined at the single cell level might seem more relevant to the biology of monocytes (Table 2). Alternatively, since monocytes are innate, non-clonal immune cells with limited functions inside the vasculature aside patrolling of the endothelium by non-classical monocytes^11^, functional relevance may depend on the expression of discrete molecules in cells which otherwise express disparate phenotypes. Adhesion molecules would be candidates for such a role given their involvement in cell recruitment and tissue infiltration. Thus, phenotypic groups defined according to expression of specified adhesion molecules may associate with particular cell functions. *In vitro* functional studies and analysis in patients should help determine the associations between the classifications proposed here and functional significance in different settings.

### Profiles of expression of transcription factors in human monocyte subpopulations

To further determine the identity of monocyte subpopulations, the expression of selected transcription factors was examined by qRT-PCR in purified monocyte populations obtained from three healthy donors. Factors that had been shown to discriminate between currently defined monocyte populations were selected^9^. These included CREB5 for its strong expression in classical monocytes, HES4 and MXD3 for nonclassical monocytes, and HES1 and EGR1 for intermediate ones^9^. NR4A1, which has been shown to be preferentially expressed in CD14^dim^CD16^+^ monocytes^27^ was also included. SPI1 (PU.1), which is strongly expressed in precursor and circulating myeloid cells^28^, was used as a positive control.

**Figure 6:**
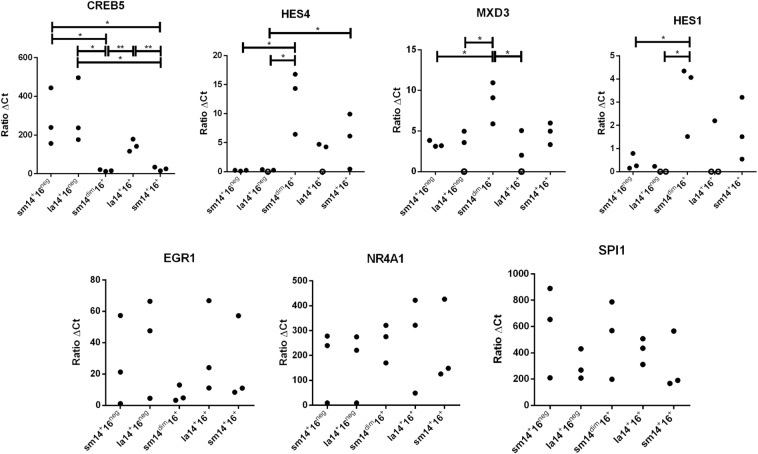
Differential expression of selected transcription factors in monocyte subpopulations. PBMC (n = 3) were isolated, stained, and analysed as described in Fig. 1, and sub-populations were purified by cell sorting. Purified cells were lysed, and RNA was purified and used as a template for cDNA synthesis. Samples were probed for the quantitative expression of indicated genes in a Taqman expression system. Data were expressed using the 2^−ΔΔCt^ method. (*p < 0.05; **p < 0.01 Mann-Whitney U test).

As shown in Fig. 6, CREB5 was strongly expressed in sm14^+^16^neg^ and in la14^+^16^neg^. This may be expected if both subsets would be considered as part of CD16^neg^ classical monocytes analysed by others^9,29^. However, it was also expressed in la14^+^16^+^ monocytes at a level similar to those of CD16^neg^ monocytes. No expression was found in sm14^dim^16^+^ and sm14^+^16^+^ monocytes.

HES4 and MXD3 were strongly expressed in sm14^dim^16^+^ monocytes (Fig. 6) that are likely to correspond to nonclassical monocytes^9^. However, expression in other subsets was also found. HES4 expression was found in other CD16^+^ monocytes (sm14^+^16^+^ and la14^+^16^+^). MXD3 was expressed in all monocyte subsets although the level of expression was significantly lower in sm14^+^16^neg^, la14^+^16^neg^, and la14^+^16^+^ monocytes.

Expression of HES1 and EGR1 was found in sm14^+^16^+^ monocytes but was also strong in other subpopulations (Fig. 6). HES1 expression was strong in sm14^dim^16^+^ monocytes and EGR1 was similarly expressed in all monocytes.

NR4A1 was also expressed in all monocytes with no significant difference between sm14^dim^16^+^ cells and other monocytes subsets in contrast to a previous report^27^.

Overall, although no difference could be detected between sm14^+^16^neg^ and la14^+^16^neg^ populations in this analysis, la14^+^16^+^ were clearly distinguished from sm14^+^16^+^ with respect to CREB5 expression.

### Cytokine production by small and large monocyte subpopulations in response to TLR agonists

**Figure 7:**
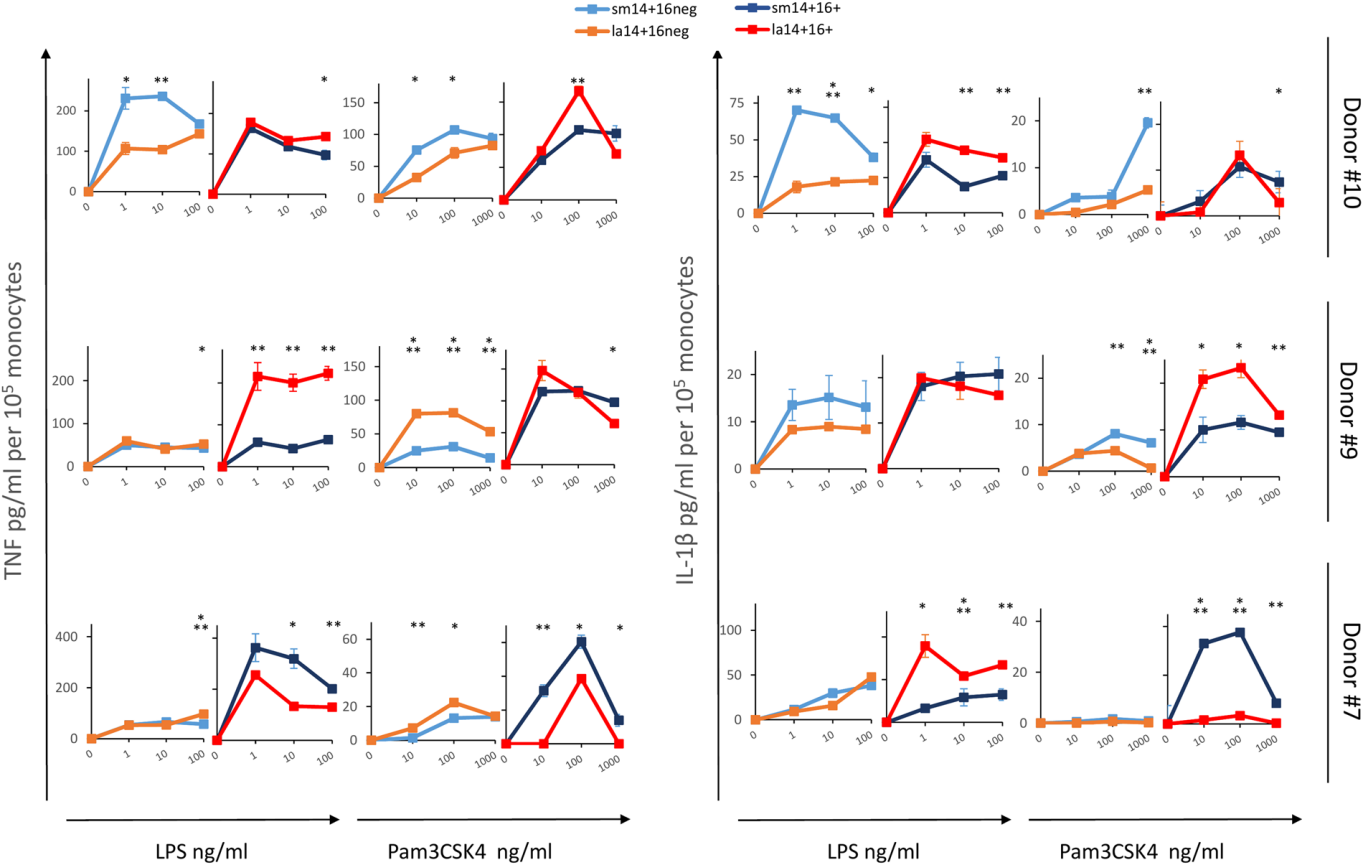
TNF and IL-1β production induced by TLR agonists in monocyte subpopulations isolated from 3 donors. Subpopulations of monocytes from donors 10, 9, and 7 (upper to lower row, respectively) were sorted and incubated overnight with increasing concentrations of LPS and Pam3CSK4, as indicated. TNF and IL-1β were assayed in supernatants by ELISA and concentrations were normalized to 1 × 10^5^ monocytes. For each condition, monocyte subpopulations with related phenotypes were grouped in a same graph: sm14^+^16^neg^ (light blue) and la14^+^16^neg^ (orange); sm14^+^16^+^ (dark blue) and la14^+^16^+^ (red). sm14^dim^16^neg^ monocytes were inconsistently detected in donors and were not included in the study; therefore, their CD16^+^ counterpart, sm14^dim^16^+^ monocytes, were not shown. Results are presented as mean and standard deviation of duplicate determinations. When error bars are not seen, they fall within the symbol. *p < 0.05, **p < 0.01, ***p < 0.001, Student’s unpaired t test.

To determine if phenotype differences between small and large monocyte subpopulations, as described above, would identify populations with distinct functions, we analysed the responses of purified subpopulations isolated from 3 donors to several TLR agonists. Cells were exposed to increasing concentrations of LPS and Pam3CSK4 (Fig. 7), and poly (I:C), imiquimod, and CpG ODN (not shown), and we assayed TNF and IL-1β present in supernatants, as well as IL-10 (not shown).

As seen in Fig. 7, although extensive diversity of responses was evident in donors, clear, dose-response dependent, statistically significant differences between small and large monocytes were found in individual donors.

Most notably, in donor 10, among CD16-negative cells, sm14^+^16^neg^ monocytes were more responsive to LPS and Pam3CSK4 than la14^+^16^neg^ monocytes (TNF and IL-1β). In CD16^+^ cells, la14^+^16^+^ monocytes produced more IL-1β in response Pam3CSK4 than sm14^+^16^+^cells. In donor 9, la14^+^16^neg^ monocytes responded more intensely to Pam3CSK4 than sm14^+^16^neg^ cells, and la14^+^16^+^ monocytes produced more TNF in response to LPS and more IL-1β in response to Pam3CSK4 compared to sm14^+^16^+^ cells. Finally, in donor 7, sm14^+^16^neg^ and la14^+^16^neg^ had minimally different responses to LPS and Pam3CSK4. In this donor, sm14^+^16^+^ monocytes produced more TNF in response to LPS and Pam3CSK4, whereas predominant production of IL-1β was alternatively found in la14^+^16^+^ monocytes (LPS) and in sm14^+^16^+^ monocytes (Pam3CSK4). Additional differences between large and small monocytes were found in responses to poly(I:C), imiquimod, and CpG ODN (data not shown).

Together, these results indicate that small and large monocytes strongly differ in their responses to inflammatory molecules. However, no clear bias toward pro- or anti-inflammatory responses were identified in small vs. large monocyte subpopulations. Differences in monocyte subpopulation responsiveness may be donor dependent. It should be noted that no unique pattern of responsiveness was also identified in CD16^neg^ versus CD16^+^ monocytes. This is not unexpected in view of the disparities of responsiveness in classical, intermediate, and non-classical monocytes reported in the literature^11–17^. Differences in monocyte subpopulations responses between donors may be associated with the phenotypic diversity of monocytes populations. Donors 7, 9, and 10 were not part of a same phenotypic group in our donor panel (Fig. 4, Tables 1 and 2).

## Discussion

The definition of human monocyte subsets has progressed by distinguishing “intermediate” CD14^+^CD16^+^ monocytes from bona fide “non-classical” CD14^dim^CD16^+^ cells within CD16^+^ monocytes^5^. However, monocyte functions often did not align with these clusters, with considerable discrepancies and overlaps in the assignment of inflammatory and immunologic roles to these subsets^11–17^. Further dissection of monocyte populations is therefore warranted in order to progress in the definition of less ambiguous functional human monocyte populations.

To advance toward this goal, we have performed a comprehensive flow cytometry analysis of multicolour-labelled human monocytes present in PBMC. First, we showed that additional populations of larger monocytes were readily detectable by flow cytometry when high numbers of events (≥1 × 10^6^) were analysed. These larger cells may have been excluded from analysis in previous studies since they can overlap with doublets in heterogeneous populations of PBMC consisting mostly of smaller cells such as lymphocytes. Here, to avoid this pitfall, we excluded doublets in populations of similar cell diameters. The larger size of these monocytes was confirmed in imaging flow cytometry. Small monocytes are seemingly included within the “classical”, “intermediate” and “non-classical” subpopulations so far described. In addition, within small monocytes, a CD14^dim^ CD16^neg^ population was also identified. Its expression of CD14, HLA-DR and CD86 together with lack of expression of lymphocyte lineage markers support its belonging to the monocyte cell type, confirming and extending results by others^30^.

Within these six monocyte populations, analysis of expression of 15 additional myeloid markers (such as FcRs, chemokine receptors, antigen presentation molecules, adhesion molecules) showed substantial variability of expression in healthy donors. Interestingly, variations in phenotype did not come out as random. Instead, groups of donors could be delineated with similar patterns of expression, and this was found in manual analysis of the data as well as in unsupervised algorithm-based analysis. Manual analysis was used to define receptor expression profiles at the subpopulation level. Combinations of these profiles defined signatures of markers expression that characterized the whole monocyte phenotype present in a donor. In addition, unsupervised analysis indicated, at the single cell level, the existence of four phenotypic groups of monocytes based on inter-individual variations of expression.

Significant differences were found between phenotypic groups defined using manual and unsupervised methods. This is however not unexpected since in manual analysis groups were coarsely defined according to mean expression of markers (mostly adhesion molecules) in whole subpopulations of monocytes. In contrast, in unsupervised analysis, combined expression of adhesion molecules at the single cell level was the basis for group definition. The significance of each of the methods of grouping will have to be determined in functional studies. A composite phenotype at the single cell level common to a group of monocytes may seem more likely to convey a specific immunologic cell function. However, taking into account the non-clonal nature of the cell type under study leads to an alternative hypothesis. Recruitment of monocytes to a tissue may depend on the expression of key cell surface adhesion molecules irrespective of the associated phenotype, and subsequent differentiation of monocytes in the tissues may follow cues from the environment^31^, superseding the original expression program present in the cells. Therefore, the differential grouping of donors using these various methods may not be competing or exclusive, and may be considered as clustering of donors according to distinct characteristics of their monocytes.

Analysis of cytokine production induced by several TLR agonists in dose-response experiments clearly showed that small and large monocytes subpopulations responded differently within donors, further suggesting that these are distinct monocyte populations. Comparison of responses between small and large populations did not reveal patterns of responsiveness, pro- or anti-inflammatory, shared by donors. Actually, within an individual donor, dominant pro-inflammatory responses were alternatively detected in small and large subpopulations depending on the TLR agonist used. For example, in donor 9 TNF responses were stronger in la14^+^16^+^ vs. sm14^+^16^+^ in response to LPS (Fig. 7), whereas the opposite was found for responses to imiquimod (data not shown); similarly, in donor 7, IL-1β production was stronger in la14^+^16^+^ in response to LPS but not in response to Pam3CSK4 (Fig. 7). Differences in signalling and/or transcriptional pathways in monocytes subpopulations may account for these opposite responses. Diversity of cytokine responses between donors was extensive and may mirror phenotype differences.

Although variability among donors in the expression of multiple genes in human monocytes has been previously described by cytofluorometry and transcriptome analysis^6,32,33^, clustering of individuals in distinct profiles of expression has not been achieved. The mechanisms leading to shared interindividual variations in monocyte phenotypes described here are unknown. Evidence has recently accumulated showing monocyte specific effects of sex and single nucleotide polymorphisms (SNP), epigenetic modifications particular to monocytes, age related alterations, as well as environmentally linked changes in monocytes (with factors such as viral infections, microbiota composition, serum lipid levels, and lifestyle choices)^34–43^.

Some of these variations have been directly linked to phenotype changes. SNPs in the *FcGR2* locus were associated with levels of CD32 expression in monocytes, but not in B lymphocytes^34^. In our results, no cluster of donors was defined based on CD32 expression, and this may be due to the smaller size or higher heterogeneity of our cohort of donors.

Sex difference has also been linked to various monocyte phenotypes and functions^35–37^. Particularly, the expression of CD163 was higher in intermediate monocytes from women^38^, a pattern however not found in this study even after dividing monocytes into subpopulations and OP profiles. In our results, although cluster sizes were small, several phenotypes were associated with sex indicated by overrepresentation of females or males in different clusters.

Age is a well-known source of variations in the immune system^35–39^. Interestingly, although our clusters included small numbers of donors, our results suggested that monocyte phenotypes might be related to the age of donors, particularly when the overall phenotype of monocyte subpopulations was considered (Supplementary Fig. S3 and Fig. 4).

Epigenetic profiles specific to individuals have been described in blood monocytes. In epigenome wide association studies (EWAS), analysis of locus specific DNA methylation signatures in classical monocytes has shown interindividual variable CpG site methylations associated with disease susceptibilities and tobacco exposure and these were specific to monocytes^39^. In bulk non-fractionated monocytes, interindividual variations in DNA methylation were linked to cis-located SNPs^40^. In addition, epigenetic modifications that were not linked to genetic variants in cis were associated with specific profiles of expression of *NFkappaB*, *CXCL8*, and *IL-10* in classical monocytes^41^.

Infections and particularly CMV status has been shown to be associated with immune cell phenotypes^36,37^. CD64 expression in classical, intermediate, and non-classical monocytes was slightly but significantly increased in CMV positive subjects^38^.

Overall, these studies suggest that multiple mechanisms are likely to affect steady-state gene expression in monocytes, and combinations of intrinsic and environmental factors may determine phenotype differences between individuals.

Complete equivalence between sm14^+^16^neg^, sm14^+^16^+^, and sm14^dim^16^+^ monocytes described in this study and classical, intermediate, and non-classical monocytes, respectively, is unlikely since CD14 and CD16 expressions in small and large monocytes partly overlapped (Fig. 1h). Small and large monocytes needed to be carefully distinguished according to their light scattering properties in order to be individualized in CD14/CD16 scatter plots. It is likely that in many previous studies classical monocytes included sm14^+^16^neg^ and parts or all la14^+^16^neg^ cells, and similarly intermediate monocytes included sm14^+^16^+^ and some la14^+^16^+^. sm14^dim^16^+^ monocytes appeared to fit with the definition of non-classical monocytes. Therefore, cell populations used previously for characterization of phenotypic or functional properties of classical and intermediate monocytes may include contaminating la14^+^16^neg^ and la14^+^16^+^ monocytes, respectively. Recently, two new populations of human monocytes have been described by RNA-seq analysis^44^, although one of them (Mono4) may correspond to contaminating NK cells^45,46^. More recently, human blood monocytes were analysed by multiparameter mass cytometry^47^. Non-classical monocytes were subdivided in three populations, and four clusters were identified in classical monocytes, one of which (population 8) may correspond to a subset of circulating dendritic cells cDC2^46^. Relationships of these populations, some of them defined according to transcriptomic profiles, with populations described here will have to be determined.

## Materials and Methods

### Donors

Blood samples from 28 healthy Caucasian donors (Supplementary Table S1) were obtained from the local blood bank in accordance with institutional regulations from the Etablissement Français du Sang, Paris, France. Written informed consent was obtained from all donors. This work was carried out in accordance with the European Code of Conduct for Research Integrity – revised edition (2017). The study was approved by the Cochin Hospital Ethics Committee (3 CCPPRB 2061) as part of an analysis of blood monocyte populations in septic patients (F. M-M, V. Faivre, A.C. Lukaszewicz, D. Payen, N. M., A. H., unpublished).

### Cell separation

Peripheral Blood Mononuclear Cells (PBMC) were isolated from venous blood collected in the presence of ACD, by density gradient centrifugation on a cushion of Ficoll as described^48^.

Briefly, blood was diluted (1:2) with sterile nonpyrogenic Phosphate Buffered Saline (PBS) and gently layered on 15 ml of Ficoll in a 50 mL Falcon tube. After centrifugation for 15 min at 800 x g at room temperature without brake, PBMC were collected and cells were washed twice (10 min, 300 × g, 4 °C) with cold PBS 1X supplemented with 10% decomplemented human AB serum (HABS_DEC_). To saturate cell surface nonspecific binding sites, cells were then incubated in 10 ml PBS supplemented with 10% HABS_DEC_ (PBS/10% HABS) for 15 min on ice. PBMC were then collected by centrifugation (5 min, 300 × g, 4 °C), re-suspended in PBS/10% HABS at a concentration of 4 × 10^6^ cells/ml, and aliquoted in FACS tubes for further use.

### Cell staining

Monoclonal antibodies (MAb) listed in Supplementary Table S2 were used. Staining of PBMC was designed for 8-color analysis with 3 sets of stained cells. Each set included lineage markers CD3, CD19, CD335/NKp46 all labelled with phycoerythrin (PE) to allow exclusion of T and B lymphocytes and NK cells, respectively. Each set also included CD14 and CD16 for monocyte subsets identification. Other markers, were unique to each set and consisted of CD64, CD32, CCR2 CCR5, CX3CR1 (set 1), CD80, CD86, HLA-DR, CD163, CD7 (set 2), and CD62L, CD162, CD43, CD49d, CD56 (set3). MAb were added to the cell suspension (2 × 10^6^ PBMC in 0.05 ml PBS/10% HABS) and samples were incubated for 20 min on ice in the dark. Optimal staining by MAbs was determined in preliminary titration experiments. Cells were then washed once in PBS/10% HABS (5 min, 300 × g, 4 °C), and once in 1 ml of PBS supplemented with EDTA (0.5 mM) (5 min, 300 × g, 4 °C). Cell pellets were recovered in 0.2 mL PBS EDTA 0.5 mM, placed on ice in the dark and analysed immediately by flow cytometry. Cell viability was analysed with Sytox-Pacific Blue dead cell stain in separate samples in order to allow use of all fluorescence channels for analysis of expressed markers. Dead cells were in most cases undetectable and never above 0.05% of the cells.

### Flow cytometry analysis

Stained cells were analysed on a FACS Canto II flow cytometer equipped for 8-color analysis. Calibration was performed as recommended in instructions provided by the manufacturer with setup procedures using 7-color Cytometer Setup and Tracking beads and automated setup adjustments keeping variations within acceptable limits set by the manufacturer. Unstained cells, isotype controls coupled to the same fluorochrome used for the marker (except for control MAb for CD32 where Pacific Orange was used as a control for Krome Orange due to lack of availability) and single labelled cells were used to set thresholds for positivity and to correct for spillovers. Compensations were automatically set by DIVA and manually checked and adjusted.

#### Manual analysis

Manual gating was performed using FlowJo V10. Doublet identification and exclusion was achieved based on fluorescence width versus area pulse measurements^49^.

Gating of cells of interest allowed us to validate compensations for each label. Thresholds for positivity were set to exclude 99% of the label obtained with isotype controls (Ig) for each marker. Percentage of positive cells and specific mean fluorescence intensity (MFI) for each label and for each subpopulation were then determined. Specific MFI was calculated as:

$${\rm{MFI}}={{\rm{MFI}}}_{{\rm{MAb}}}-{{\rm{MFI}}}_{{\rm{Ig}}}$$

Percentage of positive cells and specific MFI were presented in bubble and colour map graphs using OriginPro (Northampton, MA). MFI were designated as dim, med, and bri (for dim, medium, and bright, respectively) according to the intensity of expression of a marker in a population. Percentage of positive cells were noted as lo, int, and hi (for low, intermediate, and high, respectively) to reflect the proportion of cells that were positive for a marker in a population. MFI and percentage of positivity were written as subscript and superscript, respectively (${{\rm{X}}}_{{\rm{MFI}}}^{{\rm{percent}}\,{\rm{pos}}}$), when both were assigned to a marker. Network visualization of associations of sub-group phenotypes in donors was performed using Gephi version 0.9.1^23^. In Gephi, the layout map was obtained using the ForceAtlas 2 algorithm with repulsion and attraction strengths set at 1 000 and 1.0, respectively^24^.

#### Unsupervised analysis

Unsupervised multidimensional analysis was performed using two algorithms.

SPADE (Spanning tree Progression of Density normalized Events)^25^ was used as provided in the Cytobank suite (https://premium.cytobank.org/cytobank/login*)*. SPADE is an unsupervised data analysis algorithm, which organizes cells into hierarchies of related phenotypes (trees) and identifies population clusters. The number of target nodes was set to 600, and the size of downsampled events target was set to 10,000. Identification of populations of interest in SPADE allowed us to characterize and to define clusters of monocytes through single cell measure of 11 simultaneous parameters (FSC-A, FSC-H, FSC-W, SSC-A, SSC-H, SSC-W, CD14, CD16, CD3-CD19-NKp46, Density and Cluster).

viSNE (visualization of high-dimensional single-cell data)^26^ was also used in the Cytobank suite (https://premium.cytobank.org/cytobank/login*)*. viSNE analysis was applied to SPADE branches that included small and large monocytes.

### Imaging flow cytometry

Imaging flow cytometry was performed on a two-camera Amnis Image Stream X Mark II with INSPIRE acquisition software (Merck, Darmstadt, Germany). All images were captured with a 20x magnification objective. All cells within a gate were analysed.

Shape analysis of events used the aspect ratio feature (IDEAS software, Amnis/Millipore Corp.), and ratios between minor and major axis of each event was calculated in defined gates.

For size determination, we created a mask (IDEAS software) that traces the boundaries of cells according to CD14 expression in the image of each cell analysed. The area defined within this mask was directly related to the cell size determined with the Area Feature, and diameters were calculated from circles that had the same area as the cells (Diameter Feature, IDEAS software).

### qRT-PCR

Monocytes were isolated from PBMC by cell sorting on a FACS Aria Cell Sorter II. Cell sorting was based on negative selection against CD3, CD19 and NKp46, in order to exclude T cells, B cells and NK cells, respectively, and on the expression of CD14 and CD16.

Purified cells were lysed in TriReagent and stored at −20 °C. RNA was purified by chloroform extraction and isopropanol precipitation. RNA was dissolved in 10 µL RNAse-free water and stored at −80 °C or directly used for a reverse transcription. Quality and quantity of RNA samples were assessed using a NanoDrop 2000 Spectrophotometer.

RNA samples were used as a template for cDNA synthesis using the kit SuperScript III First-Strand Synthesis system kit and random hexamers provided by the kit were used as primers (Invitrogen, Thermo Fischer Scientific).

The quantitative expression of early growth response 1 (EGR1), cAMP responsive element binding protein 5 (CREB5), spleen focus forming virus proviral integration oncogene (SPI1), hairy and enhancer of split 4 (HES4), actin beta (ACTB) and 18 s ribosomal RNA (18S) genes were measured using the kit Takyon™ Low Rox Probe MasterMix dTTP Blue (Eurogentec, Seraing, Belgium). The necessary primers and probes (Applied Biosystems, Thermo Fischer Scientific) for qPCR use the Taqman technology, or probe hydrolysis, and were labeled at the 5′-end by FAM fluorophore and a NFQ quencher at the 3′-end. qPCR was achieved by following the recommendations of Takyon™ kit, using a 7500 Real-Time PCR system (Applied Biosystems). The sequences recognized by the probes were:

EGR1: TGACCGCAGAGTCTTTTCCTGACAT;

CREB5: TTGATGCCAATGGAGCGACAAATGT;

SPI1: CAGTCTTGGCCACCAGGTCTCCTAC;

HES4: CAGGTGACGGCCGCGCTCAGCGCCG;

ACTB: CCTTTGCCGATCCGCCGCCCGTCCA;

18S: CCATTGGAGGGCAAGTCTGGTGCCA.

Data was normalized to the expression of internal controls (ACTB and 18S) to obtain the ΔCt (Cycle Threshold). The relative quantification of the expression of mRNA was determined by using the 2^−ΔΔCt^ method.

### Monocyte activation and cytokine determinations

Monocytes sorted as described above (2–10 × 10^6^/ml in RPMI-10% FCS) were incubated overnight (37° C, 5% CO_2_) with increasing concentrations of TLR agonists. LPS O111:B4 (L-2630, Sigma-Aldrich, St Louis, MO) was re-purified by repeated phenol extractions^50^ and induced no response in TLR2 transfected HEK 293 cells. Pam3CSK4 (InvivoGen, Toulouse, France) was used as recommended by the supplier. Agonists were tested in preliminary experiments to determine optimal activation concentrations.

Supernatants were collected by centrifugation (5 min, 300 × g). Cytokine concentrations were determined by ELISA in duplicate assays using TNF and IL-1β (DuoSet, R&Dsystems, Minneapolis, MN) detection kits and were normalized to the cell number. Minimal concentrations detected were 15.6 and 3.9 pg/ml for TNF and IL-1β, respectively.

### Statistical analysis

The statistical significance of differences between subpopulations of monocytes was determined by Mann-Whitney U test (qRT-PCR, donors’ age in phenotypic clusters), confidence interval of binomial proportions (sex ratio in phenotypic clusters), one-way ANOVA (ImageStream) and Student’s unpaired t test (ELISA) using GraphPad Prism.

## Supplementary information

Supplementary information.

## Data Availability

The datasets generated during the current study are available from the corresponding author on reasonable request.
