# Supplementary material for: Identification of Novel Human Monocyte Subsets and Evidence for Phenotypic Groups Defined by Interindividual Variations of Expression of Adhesion Molecules
Source: Sci Rep. 2020 Mar 10;10:4397. doi: 10.1038/s41598-020-61022-1 (PMC7064612; doi:10.1038/s41598-020-61022-1)
Supplement: Supplementary file 1 — Supplementary information. [file 41598_2020_61022_MOESM1_ESM.pdf]

Supplementary Information

Identification of Novel Human Monocyte Subsets and Evidence for Phenotypic Groups Defined by Interindividual Variations of Expression of Adhesion Molecules

F. Merah-Mourah, S.O. Cohen, D. Charron, N. Mooney, and A. Haziot

Donor ID	Age (years)	Sex (M / F)	Monocyte fraction (%)[a]	Monocyte counts (x10^9/L)
1	33	M	5,5	0,2
2	44	M	4,4	0,2
3	42	M	5,6	0,2
4	58	M	5,2	0,2
5	40	M	4,6	0,3
6	49	M	6,3	0,3
7	37	F	5,5	0,3
8	22	F	4	0,3
9	32	M	4,1	0,2
10	62	F	5,1	0,2
11	42	F	6,6	0,3
12	33	M	4,1	0,3
13	42	M	7,8	0,3
14	29	F	6,1	0,3
15	37	F	6,7	0,4
16	29	F	5,3	0,3
17	65	M	3,7	0,1
18	26	M	6,8	0,3
19	52	F	10,1	0,5
20	61	F	5,4	0,3
21	20	M	12,7	0,5
22	55	M	6,5	0,3
23	34	F	5,8	0,3
24	46	M	5,1	0,2
25	61	F	4,8	0,2
26	63	M	9	0,4
27	42	M	3	0,2
28	41	M	6,4	0,2

Supplementary Table S1. Characteristics of blood donors

[a], numbers express the percentage in PBMC

Marker	Clone	Isotype	Clone of isotype control	Label	Marker Supplier	Isotype Supplier	Test Tube used
**CD3**	UCTH1	IgG1	MOPC-21	PE	Biolegend	Biolegend	1,2 & 3
**CD19**	HIB19						
**NKp46**	9E2						
**CD14**	RMO52	IgG2a	MOPC-173	FITC	Beckman & Coulter	Biolegend	1,2 & 3
**CD16**	3G8	IgG1	MOPC-21	PerCP-Cy5.5	BD	BD	1,2 & 3
**CD64**	10.1	IgG1	MOPC-21	APC-H7	BD	BD	1
**CD32**	2E1	IgG2a	L11511	Krome Orange / Pacific Orange	Beckman & Coulter	Invitrogen	1
**CD192/CCR2**	TG5/CCR2	IgG2b	MPC-11	Alexa Fluor647	Biolegend	Biolegend	1
**CD195/CCR5**	HEK/1/85a	Rat IgG2a	RTK2758	Pacific Blue	Biolegend	Biolegend	1
**CX3CR1**	2A9-1	Rat IgG2b	RTK4530	PE-Cy7	Biolegend	Biolegend	1
**CD80**	L307.4	IgG1	MOPC-21	APC-H7	BD	BD	2
**CD86**	2331/FUN-1	IgG1	MOPC-21	PE-Cy7	BD	BD	2
**HLA-DR**	L243/G46-6	IgG2a	G155-178	V500	BD	BD	2
**CD163**	RM3/1	IgG1	MOPC-21	Alexa Fluor647	Biolegend	Biolegend	2
**CD7**	M-T701	IgG1	X40	V450	BD	BD	2
**CD62L**	Dreg-56	IgG1	679.1MC7	APC-Alexa Fluor750	Invitrogen	Beckman & Coulter	3
**CD162**	FLEG	IgG2a	eBM2a	APC	eBioscience	eBioscience	3
**CD43**	DF-T1	IgG1	IS5-21F5	VioGreen	Miltenyi	Miltenyi	3
**CD49d**	9F10	IgG1	MOPC-21	PE-Cy7	Biolegend	Biolegend	3
**CD56**	MEM-188	IgG2a	MOPC-173	Pacific Blue	Biolegend	Biolegend	3

Supplementary Table S2. Antibodies used. Monoclonal antibodies were from mouse unless specified.

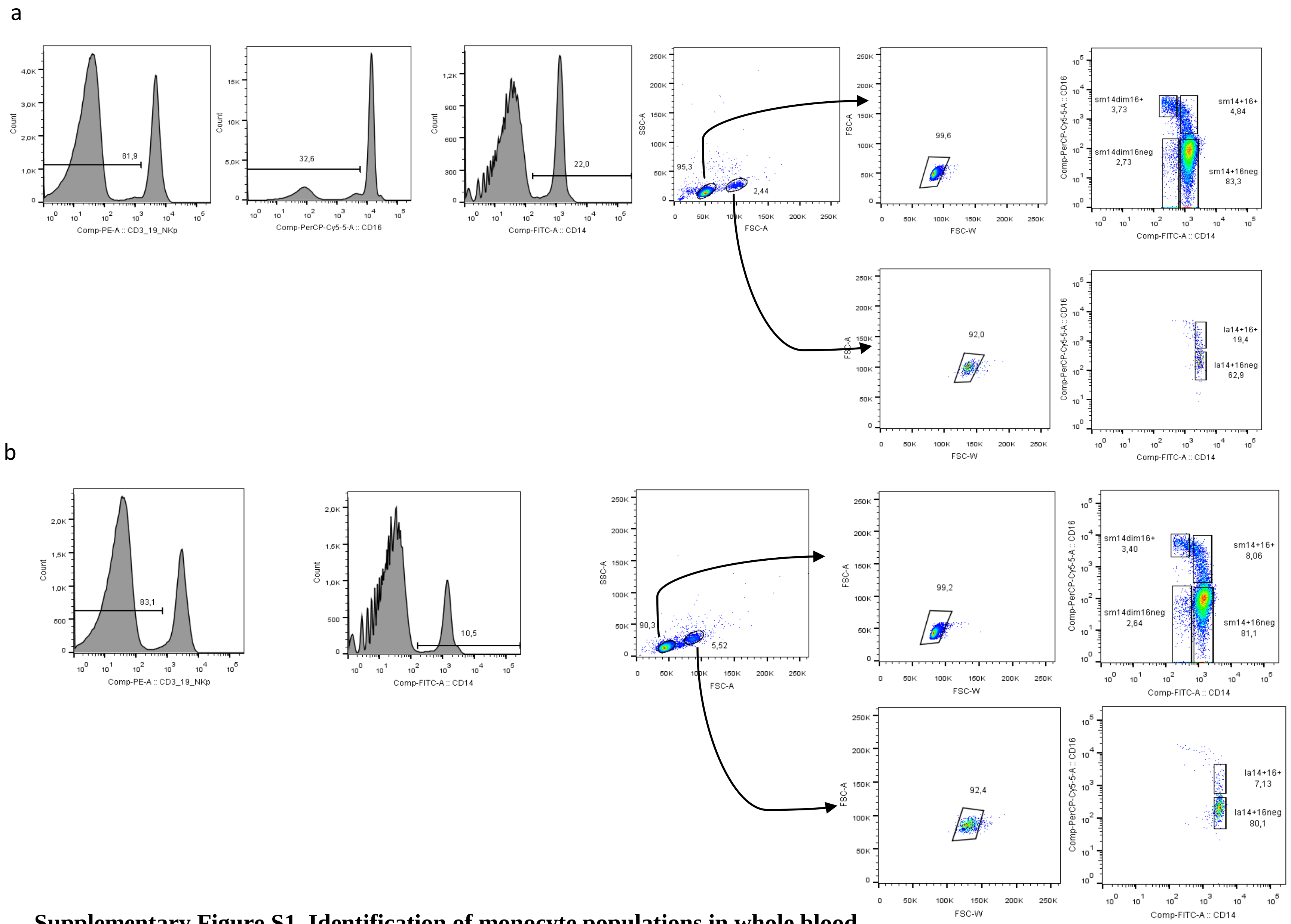

Supplementary Figure S1. Identification of monocyte populations in whole blood.
Blood cells (n = 4) were simultaneously stained in whole blood (a) and PBMC fractions (b). In whole blood, lymphocytes (CD3/CD19/NKp46-positive cells) and granulocytes ($CD16^{bright}$) (51) were excluded, and $CD14^+$ cells were selected. Small and large monocytes were identified in forward and side scatter plots. After doublet removal, CD14 and CD16 expressions were analysed. PBMC samples (b) were analysed as described in Figure 1. Data shown is from one representative individual.

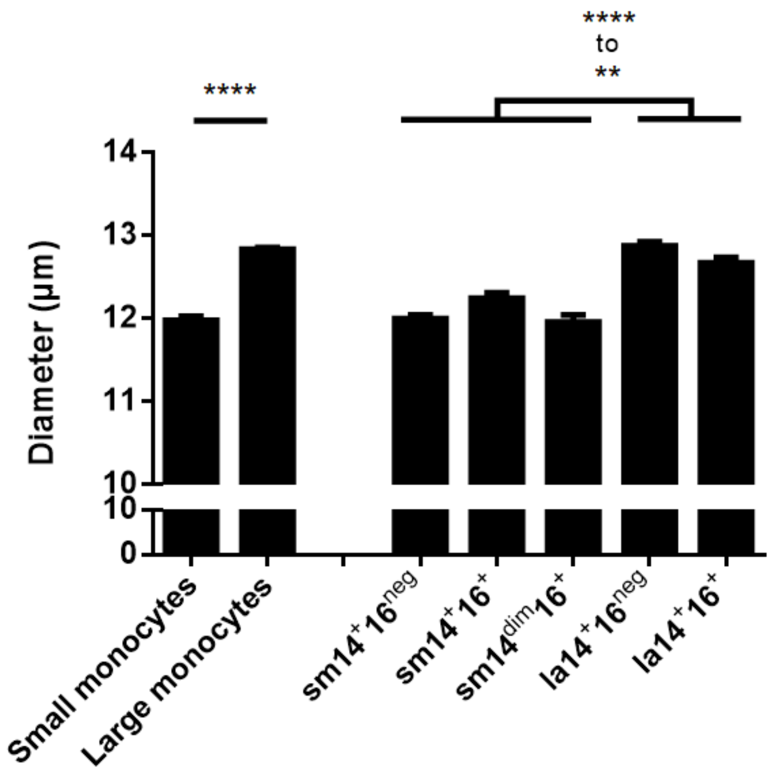

Supplementary Figure S2. Cell diameters in monocyte subpopulations determined by Imaging flow cytometry analysis.
PBMC (n = 3) were stained and analysed by imaging flow cytometry (ImageStream, Amnis). Cell diameters were determined using Diameter Feature (IDEAS software) with a mask delimited by CD14 expression. Measures were carried out in small and large monocytes, in each small monocyte subpopulation, and in each large monocyte subpopulation in 3 donors. Diameter differences between small and large monocytes (11.98 ± 0.05 vs. 12.84 ± 0.02, mean ± SEM, in µm) were significantly different (****, $p \leq 0.0001$, one-way ANOVA). sm$14^{+}16^{neg}$, sm$14^{+}16^{+}$, and sm$14^{dim}16^{+}$ monocytes had similar diameters, and large monocyte subpopulations la$14^{+}16^{neg}$ and la$14^{+}16^{+}$ had also very similar diameters. Two by two comparisons between small and large monocyte subpopulations confirmed the significance of diameter differences (**, $p \leq 0.01$ to ****, $p \leq 0.0001$, one-way ANOVA).

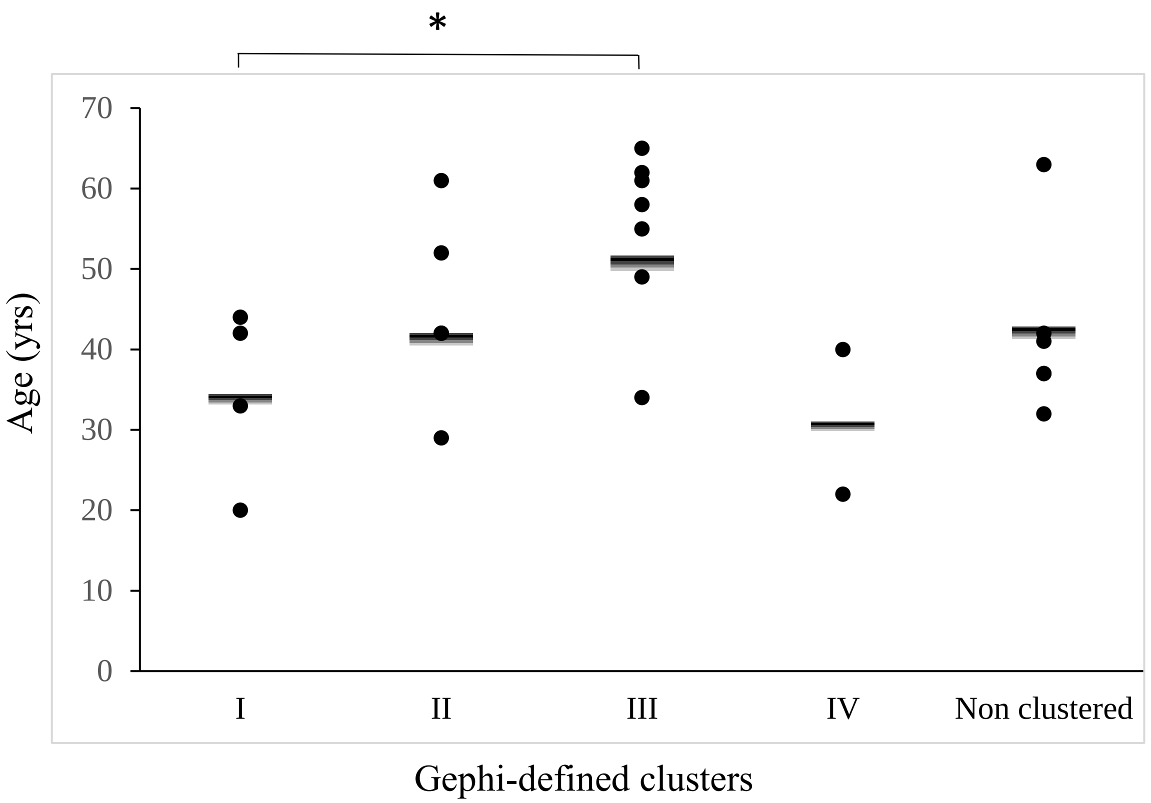

Supplementary Figure S3. Donors' age in Gephi-defined phenotypic clusters of donors (see Figure 4).
Age (in years) of donors clustering according to monocyte sub-population OP-profiles as identified with the network analysis software Gephi. Circles may represent two donors of the same age. Bars represent the mean age within the clusters. *, p = 0.045, Mann-Whitney U test.

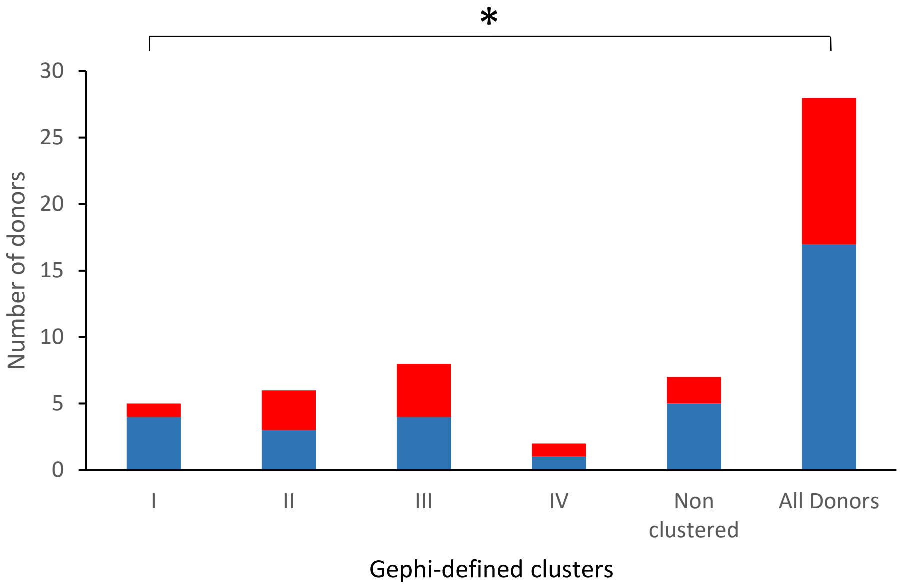

Supplementary Figure S4. Sex ratio in Gephi-defined phenotypic clusters of donors (see Figure 4).
The numbers of females (red) and males (blue) in each cluster as defined in Figure 4 and in the total population of donors is represented. *, significant difference in sex ratio between cluster I (F = 0.200 ; M = 0.800) and parent population with a 98% confidence interval (modified Wald method) (52) of [0.212 – 0.608] and [0.392 – 0.788] for females and males, respectively, in the parent population.

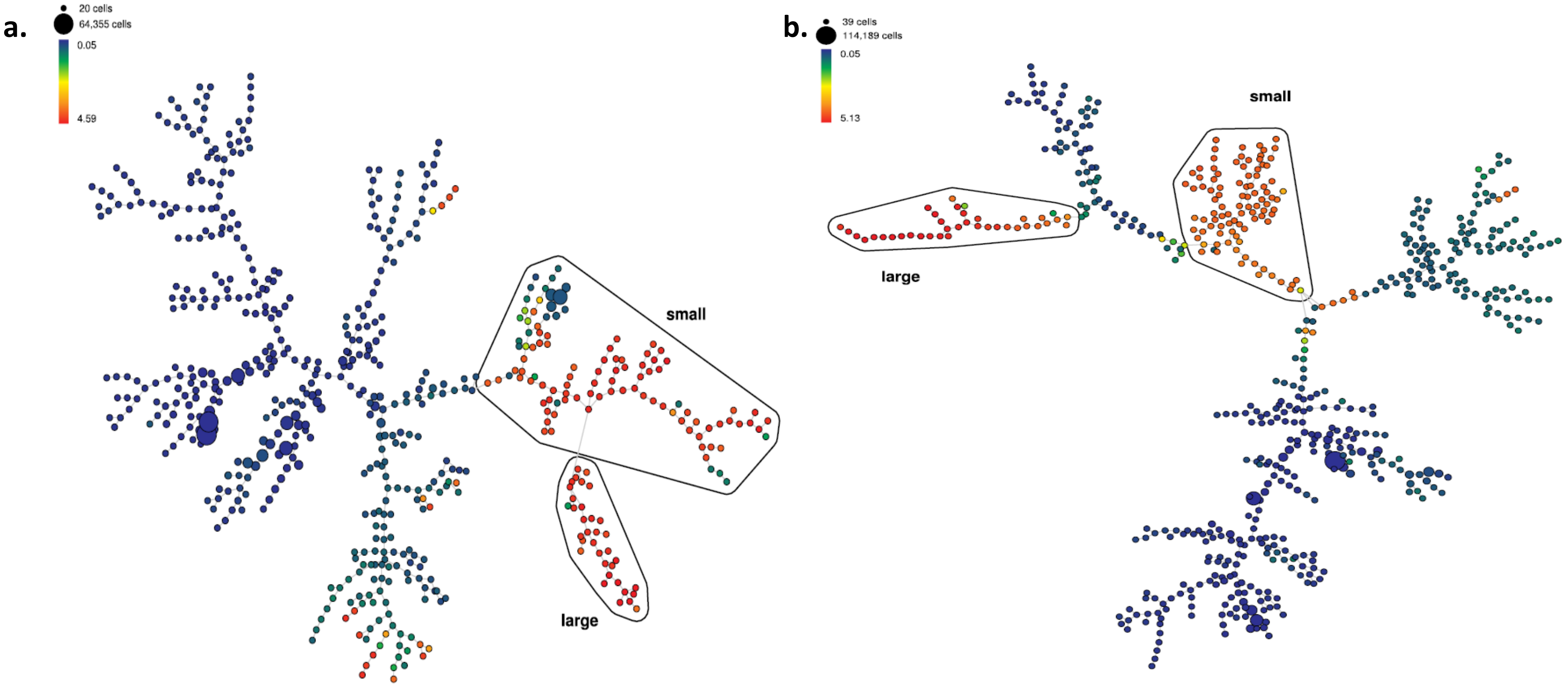

Supplementary Figure S5. Identification of small and large monocytes using unsupervised analysis tool SPADE.
PBMC were isolated and stained as described in Figures 1 and 3 and a hierarchy of related phenotypes was determined by SPADE (target number of nodes: 600; down-sampled events targets: absolute number 10,000). CD14 expression is presented according to corresponding colour scales. Gated branches were identified as small and large monocytes according to their FSC/SSC profiles. Two SPADE profiles were identified according to monocyte hierarchies: (a) profile SP-1 with representative donor 13 is shown, and (b) profile SP-2 with representative donor 4 is shown. In SP-2, a cell population of low to negative CD14 expression and low side scatter was intercalated between the branches corresponding to small and large monocytes. SP-1 and SP-2 structures were found in eleven and seven donors, respectively. Four donors had tree structures that did not fall within the SP-1 or SP-2 types and were unique to each donor. In six donors (5, 10, 11, 26, 27, and 28), small and large monocytes did not cluster in single, well-defined branches although they were readily detected in FSC/SSC plots.

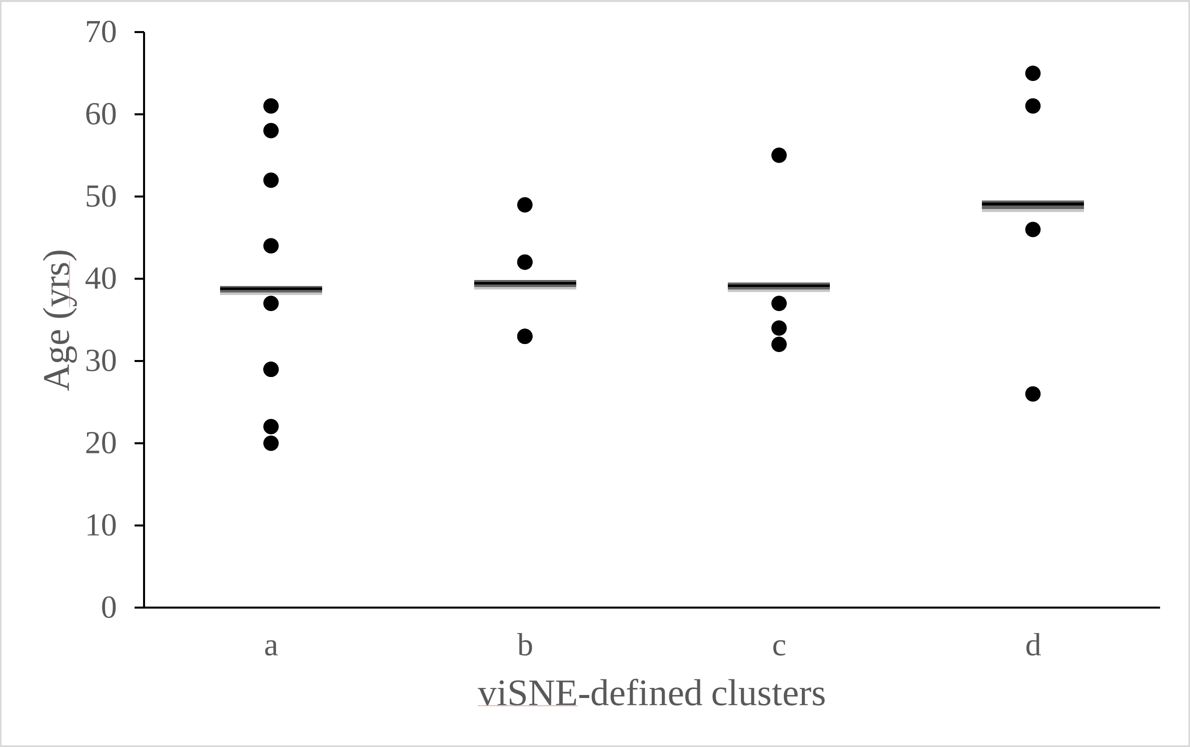

Supplementary Figure S6. Donors' age in viSNE-defined clusters (see Figure 5).
Age (in years) of donors clustering according to monocyte profiles defined by viSNE analysis. Bars represent the mean age in clusters.

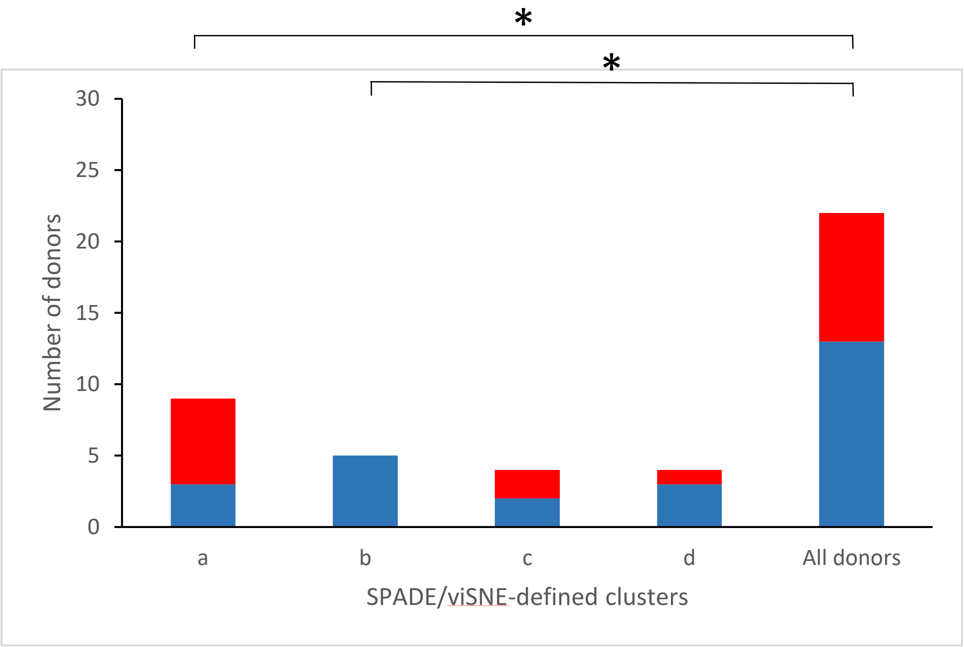

Supplementary Figure S7. Sex ratio in viSNE-defined clusters of donors.
The numbers of females (red) and males (blue) in each cluster defined in Figure 5 and in the total population of donors is represented. *, a significant difference in sex ratio between cluster a (F = 0.667 ; M = 0.333) and parent population, and cluster b (F = 0 ; M = 1) and parent population, with a 98% confidence interval (modified Wald method) (52) of [0.207 – 0.647] and [0.353 – 0.793] for females and males, respectively, in the parent population.
